# Quality of Life, Pedobarographic Parameters, and Foot Disorders in Patients with Extreme Obesity: Preliminary Results on Changes After Bariatric Surgery with Gastric Bypass

**DOI:** 10.1007/s11695-023-06843-5

**Published:** 2023-10-10

**Authors:** Ana María Pérez Pico, María Ángeles Gómez González, María Isabel Alarcón González, Julia Villar Rodríguez, Raquel Mayordomo Acevedo

**Affiliations:** 1 DEDAP Research Group, Department of Nursing, Centro Universitario de Plasencia, Universidad de Extremadura, Plasencia, Cáceres, Spain; 2 DEDAP Research Group, Department of Nursing, Centro Universitario de Plasencia, Universidad de Extremadura. Prevention Unit, Virgen del Puerto Hospital, Plasencia, Cáceres, Spain; 3 Bariatric Surgery Unit, Virgen del Puerto Hospital, Plasencia, Cáceres, Spain; 4 DEDAP Research Group, Department of Anatomy, Cell Biology and Zoology, Centro Universitario de Plasencia, Universidad de Extremadura, Plasencia, Cáceres, Spain

**Keywords:** Quality of life, Extreme obesity, Foot disorders, Pedobarography

## Abstract

**Purpose:**

Obesity is a growing health problem that affects a high percentage of the population. In podiatry context, few studies have addressed obesity because most pedobarographic systems are unable to bear the weight of patients with obesity, making it difficult to examine and manage these patients. The objective of this study was analyzed the sociodemographic characteristics, quality of life, foot disorders, and pedobarographic parameters of patients with extreme obesity who are candidates for bariatric surgery and determine the changes after weight loss post-surgery.

**Materials and Methods:**

We conducted a foot examination, a pedobarographic study using a Podoprint® pressure platform, and a quality of life questionnaire (EQ-5D) on 23 patients with extreme obesity and analyzed the changes 12–18 months after surgery in 11 of them.

**Results:**

We observed foot disorders, high plantar pressure, greater rearfoot contact, flat footprint, asymmetries, and alterations in toe contact. Almost 73.9% of participants said they had foot pain, 56.5% said they had impaired mobility, and more than 40% said they had limitations in carrying out daily activities and suffered from anxiety. After weight loss, we observed improved quality of life; more foot disorders; changes in total contact area, plantar pressures, barycenter, contact time, and footprint; decreased pain perception, walking problems and anxiety situations. Moreover, medication decreased, but they need to take more vitamins and calcium.

**Conclusion:**

Weight loss improved the quality of life of the participating patients but altered their foot disorders. All parameters need regular reassessment to detect changes and modify initially prescribed treatments.

**Graphical Abstract:**

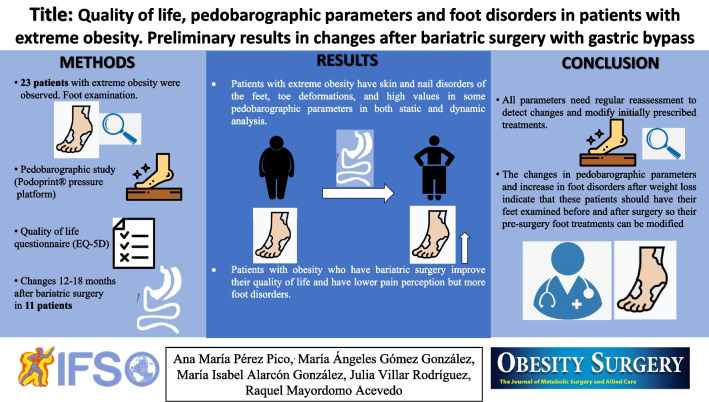

## Introduction

Obesity is defined as an excess of adipose tissue resulting in weight gain that endangers health [1, 2]. It is considered one of the most important health problems of our time and an avoidable cause of death. Extreme obesity is associated with an increase in long-term morbidity and mortality. It reduces the life expectancy of sufferers and is a growing health problem worldwide. The prevalence of extreme obesity is expected to double exponentially in the coming years [3].

The World Health Organisation considers that a person has class III obesity (extreme obesity) if their body mass index (BMI) is higher than 40 [1]. Extreme obesity is associated with diabetes, arterial hypertension, and heart disease, among other conditions [3, 4]. It can occur with mental health problems such as depression, anxiety, and other behavioral disorders [5, 6], further decreasing the quality of life of these patients.

Surgery is the final option for treating patients with severe obesity. Several types of bariatric surgery are available although one of the most frequent choices is the gastroduodenal bypass because of its considerable effectiveness in weight loss and low mortality [7, 8].

Moderate physical exercise is highly recommended to achieve the goal of reducing weight [9], It is essential to follow the procedures established by the ACSM (American College of Sports Medicine) for exercise prescription. It is also important to analyze the medical profile of each individual, and carefully evaluate the particular needs of patients before carrying out physical activity [10]. But most patients find walking painful and difficult due to excess weight or associated conditions affecting the lower limbs [11, 12].

The literature indicates the importance of multidisciplinary care to achieve weight loss in these individuals [12–14].

In collaboration with other health professionals, podiatrists can improve the quality of life of patients with obesity, helping them to increase mobility and providing a personalized diagnosis and treatment for foot disorders. However, few studies have characterized the feet of people with extreme obesity in terms of digital morphology, skin and nails. Similarly, few works have addressed baropodometric parameters or performed longitudinal studies on changes to the foot before and after bariatric surgery in patients with extreme obesity [15].

The objective of this study was analyzed the sociodemographic characteristics, quality of life, foot disorders, and pedobarographic parameters of patients with extreme obesity who are candidates for bariatric surgery and determine the changes after weight loss post-surgery.

## Materials and Methods

### Type of Study and Duration

This study is divided into two parts. On the one hand, a cross-sectional and descriptive study was carried out with 23 study participants. On the other hand, a quasi-experimental study is conducted with 11 of these patients who underwent bariatric surgery. The study was initiated in 2019 and patients were followed for 12–18 months, from that year until July 2023. All patients were examined at the same time, always in the morning and after leaving the surgery consultation.

### Sample and Inclusion Criteria

Twenty-three patients diagnosed with extreme obesity were examined at the Bariatric Surgery Unit of the Virgen del Puerto Hospital, Plasencia (Spain) and screened to select possible candidates for bariatric surgery with gastric bypass. Eleven of them were followed up throughout the weight loss process and examined before and after surgery. The project was approved by bioethics committee of the hospital (Annex 1).

Participants had to be attending the Bariatric Surgery Unit of the Virgen del Puerto Hospital, Plasencia (Spain), be aged over 18, have extreme obesity, and sign the informed consent form. The classification of the degree of obesity and the choice of patients for surgery was carried out following the 2007 SEEDO Consensus for the evaluation of overweight and obesity. The recommendations of this Consensus for the establishment of therapeutic intervention criteria were also followed [16]; the new concept of metabolic surgery [8] and the new indications for bariatric surgery guidelines [17] were taken into account.

### Procedure

Initially, an internationally validated questionnaire on patient quality of life (EQ-5D) was conducted to analyze health status (mobility, personal care, daily activities, pain/discomfort and anxiety/depression, with a 3-point Likert scale of severity) and current perceived health status using a visual scale from 0 to 10 [18, 19]. Permission was requested for the use of this questionnaire.

Patients’ feet were examined by a qualified podiatrist, recording the presence (YES) or absence (NO) of any skin alterations (keratin disorders: hyperkeratosis (HK) or heloma (HL), skin disorders, nail disorders (onychopathies), and toe deformities. A pedobarographic study was performed using a Podoprint® pressure platform (Namrol, Barcelona, Spain) [20]. Patients were assessed in static analysis (load distribution (%), pressure distribution (g/cm^2^), weight distribution (%), and total contact area (cm^2^)) and dynamic analysis (total contact time (ms), contact phases (propulsive phase/toe off) (ms)), barycenter, footprint type and symmetries, and toe contact (presence or absence). The same procedures were performed in all cases, 12–18 months after bariatric surgery.

### Statistics

For the statistical treatment of the data obtained, a data sheet of the Microsoft Office Excel 365 program is initially used and then ordered to include them in the statistical computer package SPSS 22.0 for Windows. Different types of variables are obtained: numerical variables (continuous) and categorical variables (nominal or ordinal). The analysis of the categorical variables consists of the descriptive analysis by means of tables or distribution of frequencies, and their percentages. And in the quantitative variables, the mean and standard deviation are calculated.

## Results

### Sociodemographic Results

Most of the patients examined at the clinic were women (69.6%, ratio 16:7.) Participants had the following mean values: age 42.2 years (± 11.25), height 165,09 cm (± 8.79), weight 127.18 kg (± 22.62), BMI 46.49 (± 7.03), and stage III obesity. One participant had an eating disorder, four had an allergy, and two of them experienced falls. All patients were on medication, taking two to six types of drugs, except two patients who took only one medication.

### Quality of Life Questionnaire (EQ-5D)

More than half of the patients (*n*=13) had mobility problems. Only four patients had difficulty getting up and dressing without help. In daily activities, ten patients had difficulty and one could not perform any activity. Pain and discomfort were mentioned by seventeen patients (eight indicated a high level of pain or discomfort). Anxiety and/or depression were recorded in eleven patients, one of whom reported being very anxious and depressed. The average value of the perceived state of health was 6.04 (± 2.26).

All patients lived in the north of the Extremadura region of Spain. Two were smokers and twelve said they were ex-smokers. Seventeen patients had experience with serious illness (4 personally, 10 in their family, and 3 in caring for others). Their employment situation was varied and only four were retired. Four had worked in health services as clinical assistants. Regarding academic qualifications, three said that they only knew how to read and write, and the others had studied up to primary level or middle school level and three university studies. Twelve patients performed physical activity (walking, and in one case going to the gym mainly), with a frequency of more than 2 days a week in five cases and daily exercise in the other seven.

### Results of Foot Examination Analysis

Twenty of the 23 patients had a keratin disorder and eight had a skin disorder, (mean of disorders 2.91 ±2.25). Attention is drawn to a patient who had up to seven different keratopathies in his feet. Eighteen patients had some dermatopathies in their feet (mean of disorders 1.74±1.35). Seventeen patients had some disorder in their nails (mean of disorders 1.3 ± 1.32). It was observed that one patient had up to five disorders in his toenails. Twenty-two patients had some digital alteration (mean of disorders 2.96±1.36). One patient had more than six disorders. The prevalence and location of their foot disorders are shown in Table 1.

**Table 1:** Presence of foot disorders

Keratin disorder	*n*	Skin disorder	*n*	Nail disorder	*n*	Toe deformity	*n*
Plantar HK 1st M	5	Heel fissure	2	Onychogryphosis	5	Claw toes	9
Plantar HK 2nd M	6	Mole	2	Ingrown toenail	6	Overlapping toes (infraductus)	5
Plantar HK 3rd M	7	Xerosis	14	Longitudinal ridging	5	Overlapping toes (supraductus)	7
Plantar HK 4th M	7	Varicose veins	9	Onychomycosis	2	Hallux abductus valgus	16
Plantar HK 5th M	9	Blister	1	Onycholysis	1	Curved toes	11
Plantar HK central M	5	Hyperhidrosis	2	Beau lines	4	Hallux extensus	1
Interdigital spaces HK	1	Edema	7	Leukonychia	3	Tailor's bunion	1
Plantar heel HK	12	Eczema	1	Convolute nails	3	Swan neck fingers	1
Dorsal 2nd toe HK	1	Hyperpigmentation	2	Subungual haematoma	1		
Dorsal 3rd toe HK	1	Blister	1				
Dorsal 5th toe HK	3	Mole	2				
Pinch callus	7						
Periungual HL	1						
HL 2nd M	1						
External logitudinal Arch HK	1						

*n*, number of patients with the disorder; *HK*, hyperkeratosis; *1st*, first; m, metatarsal; *2nd*, second; *3rd*, third; *4th*, fourth; *5th*, fifth; *HL*, heloma

Before surgery, all patient present four or more foot pathologies (up to 14) in their foot.

### Pedobarographic Analysis

The values of the variables measured in static analysis are shown in Table 2. Wide variability in the values is observed, with a difference of 96 kg in weight, 282 cm in contact area, 1527.5 g/cm^2^ in maximum pressure and 263.3 g/cm^2^ in mean pressure. The most frequent barycenter was posterior left. The flat footprint was the most common and several asymmetrical footprints were observed. None of the patients had ground contact with all toes. Load distribution in both the right foot (RF) and the left foot (LF) was greater in the rearfoot.

**Table 2:** Pedobarographic study of the variables measured in static analysis in patients with extreme obesity

Static baropodometric study									
*N* (*B*)	Age (years)	Weight (kg)	Contact area (cm^2^)	Maximum Prs (g/cm^2^)	Mean Prs (g/cm^2^)	Barycenter	Footprint type	Asymmetrical footprint	Alt. toe contact
TM	42.22 (±11.25)	127.8(±22.62)	300.34(±58.35)	1111.1(±532)	416.6(± 70.08)	--------	---------	---------------	----------
1B/1	32	155.0	302.00	1974.0	503.0	Left posterior	Normal	No	Yes
2B/2	46	86.7	178.00	1952.0	478.0	Right posterior	Cavus/normal	Yes	Yes
3B/3	26	145.0	296.00	1557.0	490.0	Left posterior	Flat	No	Yes
4B	40	148.0	330.0	1343.0	465.0	Posterior center	Flat	No	Yes
5B	45	143.5	315.00	1423.0	463.0	Right anterior	Normal	No	Yes
6B	40	105.7	261.00	1463.0	405.0	Posterior center	Flat	No	Yes
7B/4	47	102.7	260.00	1433.0	422.0	Left posterior	Flat	No	Yes
8B	32	127.6	266.0	1332.0	449.0	Left posterior	Flat	No	Yes
9B	49	113.2	262.0	1345.0	431.0	Left posterior	Flat	No	Yes
10B	41	126.0	251.0	1657.0	502.0	Right posterior	Flat	No	Yes
11B	31	113.0	239.0	1350.0	447.0	Right posterior	Normal	No	Yes
12B	22	136.0	254.0	1786.0	521.0	Posterior center	Flat	No	Yes
13B	56	134.0	317.0	1418.0	423.0	Right posterior	Flat/cavus	Yes	Yes
14B/5/	44	176.0	400.00	602.80	440.00	Posterior center	Flat	No	Yes
15B/6	40	121.0	380.00	446.50	318.40	Left Posterior	Flat	No	Yes
16B	66	80.0	281.00	503.60	284.70	Left posterior	Flat	No	Yes
17B/7	34	125.0	313.00	538.70	399.40	Right center	Cavus	No	Yes
18B	35	110.0	305.00	568.60	306.70	Right center	Cavus	No	Yes
19B/8	52	136.0	293.00	639.40	464.20	Left Posterior	Flat	No	Yes
20B	37	146.0	291.00	730.00	316.00	Left posterior	Flat	No	Yes
21B/9	60	111.0	311.00	468.10	347.30	Right Posterior	Flat	No	Yes
22B/10	36	133.0	343.00	520.70	379.00	Right Posterior	Flat	No	Yes
23B/11	60	151.0	460.00	504.00	328.30	Right center	Cavus	No	Yes
Static pedobarographic study									
*N* (*B*)	Load distribution forefoot LF (%)		Load distribution forefoot RF (%)	Load distribution rearfoot LF (%)	Load distribution rearfoot RF (%)	LF force borne (%)	RF force borne (%)	LF weight borne (kg)	RF weight borne (kg)
TM	38.57 (± 10.21)		39.74 (± 9.35)	62.11 (± 11.8)	59.73 (± 10.15)	47.53 (± 6.75)	52.33 (± 6.83)	61.31 (± 14.76)	65.61 (± 12.48)
1B/1	32		43	68	57	54.00	46.00	84	71
2B/2	36.0		38.0	64.0	62.0	25.0	75.0	21.0	64.0
3B/3	44.0		38.0	56.0	62.0	59.0	41.0	86.0	59.0
4B	38.0		31.0	62.0	69.0	48.0	52.0	71.0	77.0
5B	60.0		47.0	40.0	53.0	40.0	60.0	57.0	87.0
6B	20.0		22.0	80.0	78.0	54.0	49.0	54.0	52.0
7B/4	35.0		46.0	65.0	54.0	49.0	51.0	50.0	52.0
8B	33.0		37.0	67.0	63.0	51.0	49.0	65.0	62.0
9B	30.0		36.0	70.0	64.0	49.0	51.0	56.0	57.0
10B	21.0		26.0	79.0	74.0	40.0	60.0	50.0	76.0
11B	39.0		38.0	61.0	62.0	49.0	51.0	56.0	57.0
12B	18.0		19.0	82.0	81.0	50.0	50.0	69.0	67.0
13B	47.0		43.0	53.0	34.0	40.0	60.0	53.0	81.0
14B/5/	41.80		40.80	58.20	59.20	48.60	51.40	85.50	90.50
15B/6	38.00		36.80	62.00	63.20	49.50	50.50	59.90	61.10
16B	47.20		43.30	52.70	56.70	52.20	47.80	41.80	38.20
17B/7	46.20		49.40	53.80	50.60	46.80	53.20	58.50	66.50
18B	48.00		46.40	52.00	53.60	50.50	49.50	55.50	54.50
19B/8	46.80		43.50	53.20	56.50	40.50	59.50	66.90	69.10
20B	38.00		41.00	62.00	59.00	50.00	47.00	75.00	71.00
21B/9	48.50		60.80	51.50	49.20	52.00	48.00	56.20	51.80
22B/10	46.60		51.10	53.40	48.90	49.50	50.50	65.80	67.20
23B/11	33.20		36.00	66.80	64.00	48.80	51.20	73.70	77.30

*N*, patient number; *B*, pre-surgery measurements; *kg*, kilograms; *cm*^*2*^, square centimeters; *Prs*, pressure; *g/cm*^*2*^, grams per square centimeter; *alt*, alteration; *TM*, mean value of total sample; *LF*, left foot; *RF*, right foot; *%*, percentage

The dynamic measurement results are shown in Table 3. Total foot support in most patients is similar in both feet, whereas in the propulsive period, the highest value is mostly in the RF. A high number of patients did not perform toe off, taking off on the central metatarsals instead. It was observed that four patients did not do the digital take off on one foot. Two individuals were flat-footed, with no propulsive period or toe off.

**Table 3:** Pedobarographic study of dynamic variables

Dynamic baropodometric study						
*N* (*B*)	Total support LF (ms)	Total support RF (ms)	Propulsive period LF (ms)	Propulsive period RF (ms)	Toe off LF (ms)	Toe off RF (ms)
TM	988.43 (± 50.54)	991.17 (± 49.49)	240.860 (± 134.83)	279.3(± 145.37)	16.69 (± 20.45)	16.04 (± 31.56).
1B/1	1000.0	1000.0	200.0	233.0	No	17.0
2B/2	1000.0	1000.0	200.0	217.0	No	No
3B/3	1000.0	1000.0	167.0	183.0	No	No
4B	1130.0	1030.0	260.0	190.0	70.0	10.0
5B	1000.0	1000.0	250.0	367.0	No	No
6B	1000.0	1000.0	483.0	350.0	No	No
7B/4	983.0	1000.0	367.0	333.0	33.0	No
8B	1000.0	1000.0	67.0	No	No	No
9B	1090.0	1090.0	440.0	420.0	50.0	50.0
10B	917.0	917.0	300.0	400.0	17.0	17.0
11B	900.0	900.0	310.0	320.0	30.0	20.0
12B	1000.0	1000.0	167.0	567.0	17.0	17.0
13B	1000.0	1000.0	No	No	No	No
14B/5/	1000.0	1000.0	107	569.00	19.00	16.00
15B/6	1000.0	1000.0	10.00	120.00	15.00	13.00
16B	984.0	1000.0	366.00	332.00	32.00	No
17B/7	941.0	950.0	392.00	339.00	11.00	19.00
18B	938.0	951.0	397.00	345.00	9.00	18.00
19B/8	998.0	1000.0	165.00	180.00	1.00	2.00
20B	920.0	920.0	151.00	240.00	60.00	150.00
21B/9	998.0	998.0	180.00	189.00	2.00	No
22B/10	990.0	990.0	181.00	188.00	3.00	1.00
23B/11	945.0	951.0	380.00	338.00	15.00	19.00

*N*, patient number; *B*, patient pre-surgery measurements; *LF*, left foot; *ms*, milliseconds; *RF*, right foot; *TM*, medium value of total sample

### Sociodemographic Results of Patients Before and After Bariatric Surgery

Analysis of the sociodemographic characteristics of the 11 patients (8 women and 3 man) who had bariatric surgery showed a mean pre-surgery weight of 131.12 kg (± 25.55). After bariatric surgery, the mean weight was 97.06 kg (± 25.38), indicating a weight loss of 34.06 kg. BMI was 47.39 kg (± 7.86) before surgery but decreased considerably after surgery (35.38 ± 9.25 kg). Despite the weight loss, 3 patients were still cataloged with obese grade II and one patient with obesity grade III. One patient became normal weight, and the degree of obesity was overweight in the rest of patients. Medication was reduced after surgery and patients stopped taking hypolipidemic agents, antidepressive, antidiabetics, antihypertensive, diuretics, and analgesics. On the other hand, it increased the intake of vitamins and calcium.

### Quality of Life Questionnaire (EQ5) Before and After Bariatric Surgery

The results of the EQ-5D questionnaire before and after bariatric surgery showed that the level of anxiety decreased in four patients, mobility problems and pain in five patients, and nine of the patients perceived an improvement in health status and two maintained it (Table 4).

**Table 4:** Results of the EQ-5D questionnaire analyzing quality of life in patients before and after bariatric surgery

EQ-5D quality of life questionnaire										
Patient (B/P)	Dif. walking	Dif. self-care	Dif. daily act.	Anxiety	Exp. serious illness	Pain	Job	Level of studies	Sport Actv.	Perceived health S.
1B/1	No	No	No	Moderate A/D	No	Moderate	Gardener	Primary	Walk 1h/7d.	8
1P	No	No	No	No	Angina	NO	Gardener	Primary	Walk 1h/7d	10
2B/2	No	No	No	No	Relative	Moderate	Clinical assistant	Secondary	Walk 1.5h/7d	7
2P	No	No	No	No	Relative	No	Clinical assistant	Secondary	Walk 1.5h/7d	8
3B/3	No	No	No	Moderate A/D	Relative/other	Moderate	No	Primary	Walk 30 min/4d	7
3P	No	No	No	No	Relative/other	No	No	Primary	Walk 1h/6d	8
7B/4	Yes (parcial)	No	No	No	Relative	Moderate	No	Secondary	No	7
7P/4	No	No	No	No	Relative	Moderate	No	Secondary	No	7
14B/5	Yes (parcial)	No	Yes (parcial)	Moderate A/D	Myself	Yes++	No	Primary	No	3
14P/5	No	No	Yes (parcial)	No	Other	Moderate	No	Primary	Walk 1.5h/2d	10
15B/6	Yes (parcial)	No	Yes (parcial)	No	No	No	Caregiver	Secondary	Walk 1.5h/2-3d	7
15P/6	No	No	No	No	No	No	caregiver	Secondary	Walk 1.5h/7d	9
17B/7	No	No	No	Moderate A/D	Relative	No	No	Secondary	Walk 1.5h/7d	4
17P/7	No	No	No	No	Myself	No	Caregiver	Secondary	Walk 1.5h/7d	10
19B/8	Yes (parcial)	Yes (parcial)	Yes (parcial)	Moderate A/D	Relative/other	Yes ++	No	Secondary	No	4
19P/8	No	No	Yes (parcial)	Moderate A/D	Relative/other	Moderate	No	Secondary	No	9
21B/9	Yes (parcial)	No	Yes (parcial)	Moderate A/D	Relative/other	Moderate	No	Secondary	Walk 1.5h/7d	4
21P/9	Yes (parcial)	No	Yes (parcial)	No	Relative	Moderate	No	Primary	Walk 1.5h/7d	8
22B/10	Yes (parcial)	No	No	No	myself	Moderate	Cleaner	Secondary	Walk 1.5h/2–3d	8
22P/10	No	No	Yes (parcial)	No	Relative	Moderate	No	Secondary	Walk 1.5h/7d	10
23B/11	No	No	No	Moderate A/D	Myself	Yes++	Caregiver	Secondary	Walk 1.5h/7d	7
23P/11	No	No	No	No	Myself	No	Caregiver	Secondary	Walk 1.5h/7d	8

Questionnaire (EQ5). *B*, patient’s pre-surgical measurements; *P*, patient’s post-surgical measurements; *Dif*., difficulty; *Exp*., experience; *Act*., activity; *S*., status; *A/D*, anxious or depressed; *h*, hours; *d*, days; *km*, kilometers; *min*., minutes

### Results of Patient Foot Examinations Before and After Bariatric Surgery

Analysis of the patients' skin showed that nine of them had a keratin disorder both before and after surgery and one developed this disorder after surgery, although the number of affected areas decreased after surgery. Specifically, hyperkeratosis in the 1st, 2nd, 3rd, and 4th metatarsals and the pink callus. It was observed that HK increases in the proximal metatarsophalangeal joint, in the heels, and in the 5th finger in the dorsal area.

Nine patients had dermatopathies before surgery and eleven after surgery. Xerosis was the pathology that increased the most, while varicose veins decreased in three patients, cracks in two, and eczema, edema, and hyperpigmentation in one patient.

Ten patients had a nail disorder before and eleven after surgery, with a decrease in stretch marks and ingrown toenails after surgery. Ten patients had a toe deformity before surgery, which remained after surgery. One patient developed a hammertoe and increased overlapping fingers, rotated fingers, and hallux abductus valgus after surgery.

After surgery, 72.72% of patient maintained or increased the number of foot pathologies. Even though a 27.28% of patient that when under bariatric surgery decrease the number of foot pathologies, they still maintained a considerable number of them.

### Baropodometric Study Before and After Bariatric Surgery

Static analysis shows that after bariatric surgery, the total contact area had decreased in almost all patients except one and only three of them did not vary the barycenter. Three patients showed a decrease in maximum pressure and ten decreased mean pressure. The footprint varied in eight patients, becoming more arched or normal. The asymmetry of the plantar footprints disappeared after surgery in one case and appeared in another. All patients presented alterations in digital support. Foot-supported force decreased in the LF after surgery in five patients and increased in six patients, while the RF-supported force increased in five patients and decreased in six patients after surgery. The weight supported by the feet decreased in three patients (2 in the LF and 1 in the RF) while it increased in two patients in the RF. The distribution of the weight supported in the feet varied in six patients after surgery, receiving more weight the LF in four cases and the RF in two. The distribution of forefoot and rearfoot loads with or without surgery showed that the rearfoot receives mostly greater loads in patients (Table 5).

**Table 5:** Pedobarographic study of static measurements before and after surgery

Static pedobarographic study									
Patient (B/P)	Age (years)	Weight kg	Contact area cm^2^	Maximum Prs (g/cm^2^)	Mean Prs (g/cm^2^)	Barycenter	Footprint type	Footprint asymmetry	Alt. toe contact
1B/1	32	155.0	302.0	1974.0	503.0	Left posterior	Normal	No	Yes
1P	34	99.0	184.0	2292.0	538.0	Right posterior	Cavus	No	Yes
2B/2	46	86.7	178.0	1952.0	478.0	Right posterior	Cavus/normal	Yes	Yes
2P	47	61.0	152.0	1675.0	401.0	Right center	Cavus	No	Yes
3B/3	26	145.0	296.0	1557.0	490.0	Left posterior	Flat	No	Yes
3P	27	122.2	261.0	1530.0	467.0	Right posterior	Flat	No	Yes
7B/4	47	102.7	260.00	1433.00	422.00	Left posterior	Flat	No	Yes
7P/4	49	86.0	296.00	428.90	290.50	Right center	Normal	No	Yes
14B/5	44	176.0	400.00	602.80	440.00	Posterior center	Flat	No	Yes
14P/5	48	143.0	372.00	770.00	336.00	Left posterior	Flat	No	Yes
15B/6	40	121.0	380.00	446.50	318.40	Left posterior	Flat	No	Yes
15P/6	41	116.0	353.00	501.10	328.60	Right center	Normal	No	Yes
17B/7	34	125.0	313.00	538.70	399.40	Right center	Cavus	No	Yes
17P/7	38	70.0	233.00	730.00	300.00	Right center	Cavus/normal	Yes	Yes
19B/8	52	136.0	293.00	639.40	464.20	Left posterior	Flat	No	Yes
19P/8	56	77.0	255.00	705.00	302.00	Left posterior	Normal	No	Yes
21B/9	60	111.0	311.00	468.10	347.30	Right posterior	Flat	No	Yes
21P/9	64	78.5	262.00	752.00	300.00	Right anterior	Normal	No	Yes
22B/10	36	133.0	343.00	520.70	379.00	Right posterior	Flat	No	Yes
22P/10	40	97.0	289.00	777.00	336.00	Right posterior	Flat	No	Yes
23B/11	60	151.0	460.00	504.00	328.30	Right center	Cavus	No	Yes
23P/11	63	118.0	392.00	675.00	300.00	Posterior center	Normal	No	Yes
Static pedobarographic study									
Patient (B/P)	Load distribution forefoot LF (%)		Load distribution forefoot RF (%)	Load distribution rearfoot LF (%)	Load distribution rearfoot RF (%)	LF force borne (%)	RF force borne (%)	LF weight borne (kg)	RF weight borne (kg)
1B/1	32.0		43.0	68.0	57.0	54.0	46.0	84.0	71.0
1P/1	31.0		46.0	69.0	54.0	47.0	53.0	46.0	53.0
2B/2	36.0		38.0	64.0	62.0	25.0	75.0	21.0	64.0
2P/2	43.0		42.0	57.0	58.0	34.0	66.0	21.0	40.0
3B/3	44.0		38.0	56.0	62.0	59.0	41.0	86.0	59.0
3P/3	28.0		24.0	72.0	76.0	49.0	51.0	60.0	62.0
7B/4	35.00		46.00	65.00	54.00	49.00	51.00	50.00	52.00
7P/4	56.10		58.00	43.90	42.00	50.00	50.00	39.80	46.00
14B/5	41.80		40.80	58.20	59.20	48.60	51.40	85.50	90.50
14P/5	37.00		41.00	63.00	59.00	53.00	47.00	66.00	59.00
15B/6	38.00		36.80	62.00	63.20	49.50	50.50	59.90	61.10
15P/6	43.30		38.60	56.70	61.40	50.60	49.40	58.70	57.30
17B/7	46.20		49.40	53.80	50.60	46.80	53.20	58.50	66.50
17P/7	44.00		51.00	56.00	49.00	38.00	62.00	27.00	43.00
19B/8	46.80		43.50	53.20	56.50	40.50	59.50	66.90	69.10
19P/8	39.00		37.00	61.00	63.00	51.00	49.00	40.00	37.00
21B/9	48.50		60.80	51.50	49.20	52.00	48.00	56.20	51.80
21P/9	48.00		56.00	52.00	44.00	55.00	45.00	43.00	36.00
22B/10	46.60		51.10	53.40	48.90	49.50	50.50	65.80	67.20
22P/10	45.00		47.00	55.00	53.00	49.00	51.00	48.00	44.00
23B/11	33.20		36.00	66.80	64.00	48.80	51.20	73.70	77.30
23P/11	39.00		40.00	61.00	60.00	46.00	54.00	54.00	64.00

*Kg*, kilograms; *cm*^*2*^, square centimeters; *Prs*, pressure; *g/cm*^*2*^, grams per square centimeter; *alt*., alteration; *B*, patient’s pre-surgery measurements; *P*, patient’s post-surgery measurements; *LF*, left foot; *%*, percentage; *RF*, right foot

The dynamic study showed that total contact in both feet was unchanged in three patients and decreased in the other after surgery. The propulsion period of the LF is longer in four patients and shorter in six, and one has the same time before and after surgery. In the RF of four patients, the propulsion period is longer and in six patients it is shorter. The digital takeoff appeared in the LF in one patient and in another it disappears after surgery. In RF, digital takeoff disappeared in two patients and appeared in one after surgery (Table 6).

**Table 6:** Dynamic baropodometric study before and after surgery

Dynamic pedobarographic study						
Patient (B/P)	Total contact LF (ms)	Total contact RF (ms)	Propulsive period LF (ms)	Propulsive period RF (ms)	Toe off LF (ms)	Toe off RF (ms)
1B/1	1000.0	1000.0	200.0	233.0	No	17.0
1P	1000.0	1000.0	133.0	350.0	No	No
2B/2	1000.0	1000.0	200.0	217.0	No	No
2P	940.0	950.0	390.0	340.0	10.0	20.0
3B/3	1000.0	1000.0	167.0	183.0	No	No
3P	1000.0	1000.0	183.0	183.0	No	No
7B/4	983.0	1000.0	367.00	333.00	33.00	No
7P/4	1000.0	1000.0	350.00	83.00	No	No
14B/5	1000.0	1000.0	307.00	269.00	19.00	16.00
14P/5	920.0	920.0	150.00	240.00	50.00	160.00
15B/6	1000.0	1000.0	10.00	120.00	15.00	13.00
15P/6	1000.0	1000.0	350.00	83.00	No	No
17B/7	941.0	950.0	392.00	339.00	11.00	19.00
17P/7	870.0	860.0	180.00	180.00	30.00	40.00
19B/8	998.0	1000.0	165.00	180.00	1.00	2.00
19P/8	1000.0	870.0	220.00	210.00	10.00	30.00
21B/9	998.0	998.0	180.00	189.00	2.00	No
21P/9	940.0	950.0	180.00	120.00	50.00	10.00
22B/10	990.0	990.0	181.00	188.00	3.00	1.00
22P/10	930.0	1000.0	180.00	200.00	70.00	80.00
23B/11	945.0	951.0	380.00	338.00	15.00	19.00
23P/11	910.0	950.0	150.00	100.00	40.00	30.00

*LF*, left foot; *ms*, millisecond; *RF*, right foot; *B*, patient’s pre-surgery measurements; *P*, patient’s post-surgery measurements

## Discussion

Extreme obesity is a health problem that affects a significant proportion of the population [1] [2] [13]. It is known to be associated with multiple comorbidities, such as cardiovascular disease, diabetes, disorders of the locomotor apparatus [4], and some cancers [21] [22], and reduces quality of life [5] [23]. Some authors have reported a growing interest in the consequences of weight gain on quality of life and health [24]. Multiple factors have been analyzed in patients with obesity or extreme obesity, including mental health problems [25] [26], perceived musculoskeletal pain [27, 28], the impact of BMI on deterioration in quality of life [23], skin disorders [29], kinematic and kinetic parameters [30], and changes in balance associated with pressure center [28, 30, 31]. Kolotkin et al. [24] reported that patients with obesity undergo alterations in physical and psychosocial functioning, but most quality-of-life studies are conducted in the context of treatment rather than the general population of people with obesity. Our study analyzed a group of people with extreme obesity who came to a clinical consultation as candidates for bariatric surgery and followed up on those who had surgery. By addressing physical and psychosocial functioning, we were able to observe changes in patients after surgery, not only through questionnaires but also through the full examination of the lower limb.

The results obtained in the EQ-5D questionnaire before and after surgery show less perceived pain, in agreement with the findings of other authors [27, 28]. In addition, less anxiety and mobility problems were detected, and an increase in sports practice, improving the quality of life of patients. It was observed that after surgery, the intake of diuretics, hypolipidemic agents, antidepressive, antidiabetics, antihypertensives, and analgesics decreased, but intake of vitamins and calcium combinations increased. This could be due to the influence of weight loss on comorbidities, or food juggling [2, 32].

Although increasingly more studies have analyzed the consequences on the feet of weight loss after bariatric surgery [30, 31, 33, 29], we found no studies that characterized the feet of these patients in depth (nails, skin, foot deformities, static and dynamic analysis) or conducted a complete analysis of changes to the feet in the same individual after considerable weight loss. Some authors compared people with and without obesity, but in less detail than in this work [15].

Few references have addressed skin alterations in the foot of patients with extreme obesity [34, 35]. Our results show that skin alterations are frequent in these patients and that they change after surgery, in partial agreement with the study of Itthipanichpong et al. [35]. However, we found no works that mentioned as many of the skin alterations described in our work or reported increased xerosis after surgery [36]. Our study focuses solely on the foot and may therefore be more thorough in this regard. However, we have found authors who relate xerosis with vitamin B2 deficiency, so we consider that the increase in xerosis in this work could be due to the fact that sometimes surgery can lead to nutrition problems [34].

The decrease in HK in some areas and its appearance in others after bariatric surgery may be due to multiple factors. Weight loss decreased foot pressures and postural changes can cause HK to disappear. The appearance of HK may be due to a thinning of soft tissues or could even affect the increase in xerosis, in agreement with other authors [36], although we consider that it could also be due to the decrease in edema in the feet. Our baropodometric study is similar to that of Butterworth et al. [15], although these authors studied people with and without obesity. Our results show changes in the total contact area after considerable weight loss, as the values of this parameter decreased in all cases.

In conclusion, quality of life questionnaires and proper foot examination are useful to obtain information about patients, their context, and how they perceive their health and morbidities. This approach will allow health professionals to prescribe treatment with knowledge of the risks and rewards. Patients who had bariatric surgery showed improved quality of life, with a notable decrease in pain in most cases. Their considerable weight loss produced changes in all variables analyzed in their feet (skin, nails, bones, and pedobarographic study) and a majority of patient increase or maintained their foot pathologies. Therefore, it is important to examine the feet of these patients to prevent injury and prescribe specific treatments as a way to improve their quality of life through reassessment of foot care after bariatric surgery.
